# Going “social” to access experimental and potentially life-saving treatment: an assessment of the policy and online patient advocacy environment for expanded access

**DOI:** 10.1186/s12916-016-0568-8

**Published:** 2016-02-02

**Authors:** Tim K. Mackey, Virginia J. Schoenfeld

**Affiliations:** Department of Anesthesiology, University of California San Diego School of Medicine, La Jolla, CA USA; Global Health Policy Institute, 8950 Villa La Jolla Drive, A204, La Jolla, CA 92037 USA; Division of Global Public Health, University of California San Diego School of Medicine, La Jolla, CA USA; Moores Cancer Center, University of California San Diego, San Diego, CA USA; Joint Masters Program in Health Policy and Law, University of California San Diego School of Medicine, La Jolla, CA USA

**Keywords:** Expanded access, Compassionate use, Access to experimental drugs, Social media, Health policy, Regulatory science, Patient advocacy

## Abstract

Social media is fundamentally altering how we access health information and make decisions about medical treatment, including for terminally ill patients. This specifically includes the growing phenomenon of patients who use online petitions and social media campaigns in an attempt to gain access to experimental drugs through expanded access pathways. Importantly, controversy surrounding expanded access and “compassionate use” involves several disparate stakeholders, including patients, manufacturers, policymakers, and regulatory agencies—all with competing interests and priorities, leading to confusion, frustration, and ultimately advocacy. In order to explore this issue in detail, this correspondence article first conducts a literature review to describe how the expanded access policy and regulatory environment in the United States has evolved over time and how it currently impacts access to experimental drugs. We then conducted structured web searches to identify patient use of online petitions and social media campaigns aimed at compelling access to experimental drugs. This was carried out in order to characterize the types of communication strategies utilized, the diseases and drugs subject to expanded access petitions, and the prevalent themes associated with this form of “digital” patient advocacy. We find that patients and their families experience mixed results, but still gravitate towards the use of online campaigns out of desperation, lack of reliable information about treatment access options, and in direct response to limitations of the current fragmented structure of expanded access regulation and policy currently in place. In response, we discuss potential policy reforms to improve expanded access processes, including advocating greater transparency for expanded access programs, exploring use of targeted economic incentives for manufacturers, and developing systems to facilitate patient information about existing treatment options. This includes leveraging recent legislative attention to reform expanded access through the CURE Act Provisions contained in the proposed U.S. 21st Century Cures Act. While expanded access may not be the best option for the majority of individuals, terminally ill patients and their families nevertheless deserve better processes, policies, and availability to potentially life-changing information, before they decide to pursue an online campaign in the desperate hope of gaining access to experimental drugs.

**Electronic supplementary material:**

The online version of this article (doi:10.1186/s12916-016-0568-8) contains supplementary material, which is available to authorized users.

## Background

The widespread use of social media is changing the way that individuals and social networks interact and how they share, process, and consume health information, including about terminal illnesses [1]. Yet, as social media platforms increasingly become ubiquitous, many unanswered questions remain, regarding the impact of this technology on individual and population-based health through changes in health behavior and information seeking [2–7]. This includes the growing use of online petitions and social media campaigns in an attempt to gain access to experimental treatments [8–12]. This form of patient-driven advocacy enabled by “digital health” contrasts sharply with the traditional course of drug development and expanded access policies (also known as compassionate use) currently utilized by the industry and the U.S. Food and Drug Administration (FDA) [10, 13]. It also represents a direct response to the limitations of the current regulatory pathway and expanded access policies by patients who are desperate and have no viable treatment alternatives [14].

The phenomenon also raises several ethical and practical questions regarding how to ensure equitable distribution and access to scarce resources (i.e. the drug candidate), the possibility of potential harm/safety issues from untested treatment, and concerns that premature access could compromise the routine drug approval process [8–10, 15–18]. In exploring this controversial topic, we first describe the current regulatory framework for expanded access in the United States, discuss recent trends in state “right-to-try” legislation, and then identify and characterize case studies of expanded access online petitions and social media campaigns used by patients and their families. We conclude with a discussion of potential policy reforms to improve expanded access processes, including leveraging recent legislative attention focusing on reforming expanded access in the CURE Act Provisions contained in the proposed U.S. 21st Century Cures Act.

## Methods

### Literature selection criteria and online search strategy

In order to explore the U.S. policy environment for expanded access, we first searched PubMed (Medline) database for English language articles published between 2005 and 2015, which contained the keywords “expanded access”, “expanded access programs”, “compassionate use”, “right-to-try”, and “experimental drugs” in the title/abstract field using the advanced search function. We excluded literature reporting results, reviews, or meta-analyses of expanded access clinical studies or case reports, evaluations of expanded access programs, economic analysis of expanded access treatments, discussion about expanded access in countries outside of the United States, expanded access for non-pharmaceutical products (e.g. stem cell treatments and medical marijuana), and other literature that did not have policy relevance to the topic. The goal of this literature review was to describe the evolution and current regulatory and policy framework for expanded access in the United States.

We supplemented information from PubMed with additional sources of information by searching for keywords on Google search engine. This information included grey literature (informally published and non-peer reviewed written material), technical reports/guidance from government agencies, news reports, information from non-governmental organizations/patient advocacy groups, pending and enacted U.S. legislation on the topic, searching government databases relevant to expanded access data, and reviewing information from the FDA’s website. These additional information sources provided more up-to-date and current information on expanded access developments, policy issues, and debate, and helped to identify a preliminary set of expanded access patient case studies (as identified and reviewed below).

Further, in order to identify and characterize patient use of online petitions and social media and its possible association with expanded access treatment and policy, we also conducted structured web search queries. The keywords utilized in the literature review were also used to conduct searches directly on popular online petition sites and social media platforms in order to identify individual patient expanded access campaign case studies. We then reviewed identified case studies (limited to U.S. patients or their families) who sought expanded access to experimental treatment and coded content for information on types of platforms used, use of multimedia, number of signatures obtained (for campaigns using online petitions), category of disease addressed, type of treatment requested, and name of company/organization petitioned.

**Fig. 1:**
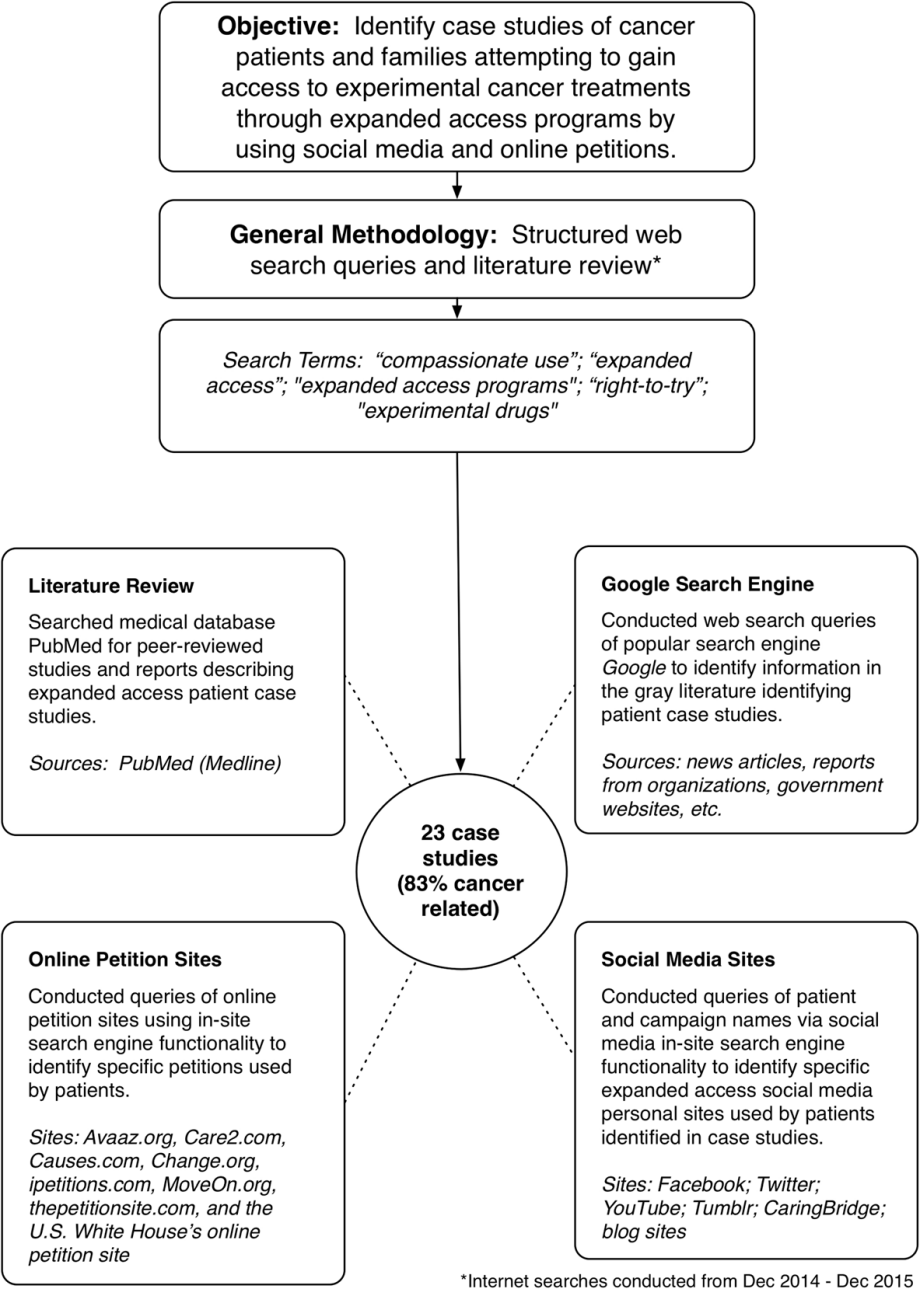
Study methodology

The literature review and structured Internet search query was conducted from December 2014–2015. A visual description of the overall study methodology is provided in Fig. 1.

## Findings

### Expanded access regulatory framework

The debate regarding expanded access is not novel to social media, with regulations first issued by the FDA in the late 1980s during the height of the HIV/AIDS epidemic and more recently becoming a topic of debate in the 2014 Ebola outbreak in West Africa [10, 19–24]. A critical turning point for patient advocacy related to expanded access occurred in 2001, when Abigail Burroughs, a terminally ill 21-year-old head and neck cancer patient, mounted a highly publicized campaign in an attempt to gain access to the then experimental epidermal growth factor receptor (EGFR) inhibitor drug Erbitux (INN: cetuximab) [25–27]. Her subsequent death in June 2001 led to the formation of the “Abigail Alliance”, an organization whose mission is to help create wider access to developmental cancer drugs and drugs for other serious life-threatening diseases, and whose efforts have led to congressional hearings, litigation against the FDA, and ultimately a landmark 2008 decision by the U.S. Court of Appeals for the District of Columbia ruling that patients did not have the constitutional right to access experimental drugs [18, 22, 26–33]. Public awareness and pressure from this event also led to a rapid growth in expanded access programs (EAPs) created by drug manufacturers, particularly for gene-targeted cancer therapeutics [34].

**Fig. 2:**
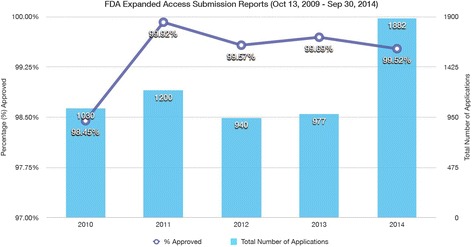
Summary of FDA Expanded Access Submission Reports (13 October 2009–30 September 2014). Source: data summarized from [42]

In 2009, substantial revisions were made to FDA policies to streamline the expanded access application process used by providers and manufacturers on behalf of patients. These revisions also included a reorganization of application categories (individual, intermediate-size populations, large populations) and sub-categories based on whether an existing investigational new drug application (IND) is available and/or whether it constitutes an emergency (defined as a life-threatening situation) [10, 35–37]. The FDA also released a 2013 draft guidance document clarifying the implementation of its 2009 expanded access process revisions and recent draft guidance in February 2015 to substantially simplify forms and speed up the process for submitting individual patient expanded access INDs [22, 38–41]. Based on data from the FDA, numerous applications are submitted each year, with the agency receiving an average of 1,206 requests annually (based on data from October 2009–September 2014) with an overall approval rate of 99 % (Fig. 2) [42–44]. The vast majority of these applications are for single-patient, non-emergency (48 %) or emergency use (44 %) INDs (versus intermediate-size patient population access programs with multiple patients with the same disease or condition seeking access to the same drug), and are assessed by the FDA on a case-by-case basis [8, 42].

Although the FDA may approve the vast majority of applications that it receives, a critical challenge to patient access continues to occur prior to FDA involvement as applicants must first obtain agreement/approval from the manufacturer/IND holder [10, 19, 43]. This approval process varies by sponsor and drug in question; many large companies have established compassionate access/EAP programs for particular drugs, but many small firms/start-ups lack any process [45, 46]. This leaves patients unable to obtain approval with few options other than appealing directly to company officials. Though firms may wish to provide an experimental drug for humanitarian, public relations, or other internal reasons, they are under no federal legal obligation to do so; indeed, courts have consistently ruled that terminally ill patients have no constitutional right to treatment with unapproved drugs [10, 19, 32, 47]. Additionally, concerns regarding reporting adverse events to the FDA arising from use in uncontrolled settings, limited drug availability, and the need to fully commit sponsors’ resources to the clinical trial and marketing approval process, may limit the willingness of firms to allow expanded access [9, 10, 32, 35, 48].

Logistics for investigational drug availability are also challenging, since these drugs are typically manufactured in small lot sizes that can be impacted by manufacturing complications and/or limited availability of active pharmaceutical ingredient/raw materials [10, 22, 32, 45, 49]. Equally challenging is the trepidation that providing patient access through expanded access channels could impact enrollment in controlled clinical trials [14, 15, 48, 50]. This is particularly the case for rare cancers or other orphan diseases, where the number of eligible study participants is limited [51–53]. Further, even if a sponsor establishes an EAP, determining the appropriate process to ensure equitable access if there is overwhelming demand can lead to difficult decisions regarding who are the best candidates to receive access and which requests to deny [8]. As an example of a response to these challenges, in May 2015, pharmaceutical manufacturer Johnson & Johnson announced a partnership with the NYU School of Medicine to establish an external advisory committee to guide its decisions on compassionate use requests [54].

### Legal and policy developments

In response to the perceived limitations of the FDA’s expanded access regulatory structure, 21 U.S.A. states have passed legislation designed to bypass the FDA’s authority to regulate experimental drug access [55, 56]. The first “right-to-try” law was passed in Colorado in May 2014, allowing patients access to experimental drugs without FDA approval and requiring only the drug sponsor’s permission, with similar laws now enacted in several other states [19, 57]. Arizona became the first state to pass a measure by public vote in November 2014 for “right-to-try” access and California is the most recent state to consider passing such legislation [58]. Generally, these various laws (mostly based on a model law drafted by the Goldwater Institute in 2014) require that patients give informed consent, have a terminal illness, have considered all other FDA-approved treatment options, have a prescription or recommendation from the patient’s physician for investigational treatment, and have medical documentation that certain requirements have been met [19, 57].

However, “right-to-try” laws have been widely criticized and uncertainty remains as to whether they can survive constitutional legal challenges [10, 19, 55]. Specifically, prior court rulings and the Supremacy Clause of the U.S. Constitution allows federal law to preempt conflicting state laws rendering them unenforceable [19, 55]. Criticism also focuses on the fact that these laws fail to address fundamental access barriers experienced by patients: primarily sponsors failing to provide compassionate use approval. Under “right-to-try” laws, participation by sponsors is voluntary, meaning that there remains no legal obligation to approve patient access to experimental drugs. The statutes may also contain rigorous informed consent requirements, but not all legislation provides adequate protection for sponsors and clinicians from potential liability, as some do not allow decisive releases of liability and others have no limitations on tort liability, likely further disincentivizing participation [19, 55, 59]. The statutes also do not require insurers to cover the cost of experimental treatments and fail to prohibit sponsors or providers from charging patients any amount they designate for activities associated with the administration of experimental treatments, potentially opening the door to exorbitant or predatory pricing to an extremely vulnerable patient population desperate for treatment [19, 55]. Another limitation to these laws is a general lack of systematic data collection and information sharing by clinicians and patients regarding treatment outcomes, even if only from an observational perspective. Due to these inherent challenges and lingering questions about federal preemption, some commentators have recommended that state “right-to-try” laws be struck down, in favor of a federal legislative policy “fix” [19, 55].

### Social media: an alternative access point?

Given the procedural and policy challenges of current expanded access pathways for investigational drugs, biologics, and medical devices, many terminally ill patients have sought alternative means for accessing treatments that are untested but may nevertheless present them with their only, albeit uncertain, chance of a life-saving intervention [8, 11, 60]. This desperate situation has given rise to patient-driven advocacy attempting to compel access to EAPs through the use of social media; a technology with multiple channels of engagement and increasing in the sphere of social and political influence [2, 6, 61].

Specifically, patients and their families use a variety of online petitions, Facebook pages, Twitter accounts, YouTube videos, and other social media platforms to raise awareness for their cause, prompt letter writing campaigns, attract traditional media coverage, and otherwise exert public pressure to obtain experimental drug access primarily targeting drug manufacturers [11, 45]. For example, compassionate use petitions facilitated by Change.org (an online petition tool hosting campaigns for individuals/organizations with over 100 million users in 196 countries) have increased from a total of three petitions in 2009 to 20 petitions in 2013 [62]. Interestingly, over the same time period, citizen petitions submitted to the FDA (searchable on the U.S. government website, www.regulations.gov) requesting changes or creation of new approval mechanisms for expanded access, have not experienced similar increases, indicating that patients may be focused more on engaging in advocacy via popularized online and social media channels [47].

In order to better understand and characterize the growing use of online petitions and social media, and its potential impact on treatment access for terminally ill patients, we conducted structured Internet search queries to identify case studies involving U.S. patients or their families who sought expanded access to experimental treatments through online campaigns. Our review yielded a total of 23 case studies, of which 87 % (n = 20) used an online petition (78 % of these used Change.org). The remaining three campaigns that did not use an online petition platform mobilized their expanded access campaign primarily using social media. The gender distribution among reviewed online campaigns was split with 52 % (n = 12) female patients compared to 48 % (n = 11) male patients and an age distribution of 61 % (n = 14) adults versus 39 % (n = 9) children subject of a petition as identified through self-reported information on websites.

The community with the highest representation among online expanded access campaigns was cancer patients and their families, who comprised 83 % (n = 19) of all case studies (which included seven pediatric cancer patients) reviewed. Terminally ill patients who used online petitions and social media suffered from a variety of cancer types, including skin, kidney, breast, brain, lung, gastric, ovarian, and soft tissue cancers. The specific cancers identified in the case studies were: stage IV melanoma (n = 4); kidney cancer (n = 2); breast cancer (n = 2); diffuse intrinsic pontine glioma (DIPG) brainstem tumor (n = 2); and lung cancer, gastric cancer, brain tumor (suspected, but not proven to be medulloblastoma), teratoma brain tumor, anaplastic medulloblastoma, stage IIIC ovarian cancer, stage IV alveolar soft part sarcoma, mantle cell lymphoma, and immunosuppression treatment for malignant rhabdoid tumor of the kidneys resulting in adenovirus (all with one patient). It should be noted that certain patient advocacy groups, such as the Multiple Myeloma Research Foundation, have actively enabled direct access to EAPs through partnerships with drug manufacturers. Hence, patients suffering from these cancer types may be less likely to pursue online campaigns given their ability to access EAPs through existing advocacy channels. Other non-cancer diseases that are extremely debilitating and life-threatening were also subject to petitions/campaigns, including spinal muscular atrophy (SMA), MPS II Hunter syndrome, coxsackievirus, and amyotrophic lateral sclerosis (ALS). Collectively these diseases comprised 17 % (n = 4) of all case studies identified.

Many of the case studies were focused on patients attempting to gain access to the same experimental drug class/therapeutic, although often for the treatment of different cancer types. As an example, 35 % (n = 8) of expanded access case studies reviewed were associated with the novel immunotherapy anti-PD-1 (a negative regulator of T-cell function that appears to recruit a much stronger immune response to tumor cells) [63]. Some of the drugs petitioned in expanded access online campaigns have also been subsequently approved by the FDA, including the Merck & Co., Inc. anti-PD-1 drug pembrolizumab (trade name Keytruda), which was approved for the treatment of non-small cell lung cancer in 2014. While several of these experimental drugs were approved for the specific indications subject to petitions (e.g. ramucirumab for advanced gastric adenocarcinoma and pertuzumab for HER2-positive metastatic breast cancer), others were approved for other treatments, indicating that even after FDA market authorization, expanded access patients may still lack access to an approved “labeled” drug for their specific condition. However, it is important to note that off-label prescribing and use for the treatment of cancer is common, and while manufacturers cannot legally promote a drug “off-label” (i.e. for indications not approved by the FDA), physicians are free to prescribe a drug for any clinical condition they see fit [52, 64, 65].

Although drug companies targeted by expanded access campaigns/petitions as a group comprised of various large, multinational global pharmaceutical firms and a few smaller mid-sized and start-up firms, Bristol-Myers Squibb and Merck & Co., Inc. were the target of the highest number of petitions due to demand for anti-PD-1 (see Table 1). Three patients also sought access to antineoplaston therapy, a highly controversial alternative cancer treatment that can be obtained in clinical trials and has also been approved by the FDA through expanded access INDs [66]. Other experimental treatments for diseases other than cancer included a petition for NurOwn™ (a form of autologous adult stem cell therapy for treatment of the neurodegenerative disease amyotrophic lateral sclerosis).

**Table 1:** Sponsor drug manufacturers subject to expanded access campaigns/petitions

Name of sponsor	Size of company^a^	Publicly traded? (Yes/No)	Number of requests^b^
BioMarin Pharmaceutical Inc.	Large	Yes (NASDAQ Stock Market: BMRN)	1
Bayer AG	Large	Yes (Frankfurt Stock Exchange: BAYN)	1
Bristol-Meyers Squibb	Large	Yes (New York Stock Exchange: BMY)	5
Chimerix Inc.	Small	Yes (NASDAQ Stock Market: CMRX)	1
CureTech Ltd.	Small	No	1
Eli Lilly and Company	Large	Yes (New York Stock Exchange: LLY)	1
Genentech Inc. (wholly owned subsidiary of F. Hoffmann-La Roche)	Large	Yes (SIX Swiss Exchange: ROG; OTCQX: RHHBY)	4
ISIS Pharmaceuticals Inc.	Small	Yes (NASDAQ Stock Market: ISIS)	1
MedImmune, LLC (wholly owned subsidiary of AstraZeneca)	Large	Yes (London Stock Exchange, OMX, New York Stock Exchange: AZN)	1
Merck & Co., Inc.	Large	Yes (New York Stock Exchange: MRK)	8
Pfizer Inc.	Large	Yes (New York Stock Exchange: PFE)	1
Pharmacyclics LLC	Large	Yes (NASDAQ Stock Market: PCYC)	1
Shire Plc	Large	Yes (London Stock Exchange: SHP; NASDAQ Stock Market: SHPG)	1
Total number of firms: 13			Total number of requests: 27

^a^Businesses classified as small (<500 employees) according to the U.S. Small Business Administration (under NAICS Association classification 541711, Research and Development in Biotechnology); ^b^total number of requests equal more than the number of cases reviewed (23), because several patients requested access from more than one sponsor

The number of signatures obtained by online expanded access petitions reviewed (i.e. an indication of outreach/public engagement) varied widely from a high of 525,738 (patient with stage IV melanoma) to a low of 448 (patient with lung cancer) [67]. Observed characteristics of “successful” case studies (defined as those that garnered greater than 100,000 signatures in online petitions) shared similar communication and outreach strategies including: 1) use of an online petition combined with message propagation through active engagement on Facebook and Twitter; and 2) use of an additional communication platform to better articulate the personal struggle of the patient (e.g. through the use of a personal website, blog, or YouTube video patient testimonial). In contrast, campaigns that received fewer signatures (i.e. 20,000 or less) often relied solely on the online petition site to raise support or only engaged the public with one additional communication medium (i.e. a blog or Facebook page). Specifically, several of the high-profile case studies reviewed which received significant traditional media coverage, appeared to have a multimedia strategy in place that was well-articulated, professionally executed (including various multimedia assets), and included coordinated message propagation across multiple popular online platforms, such as Facebook, Twitter, YouTube videos, blog sites, Tumblr, and CaringBridge.com (a social media patient support website) in addition to personal websites (see Additional file 1: Table S1 for a summary of all campaigns) [14, 68]. However, achieving robust public engagement and media coverage did not appear to associate with better chances of accessing experimental treatment through EAPs, as many of the “successful” case studies reviewed self-reported not gaining access to the treatment desired.

Despite variation in diseases addressed, treatment sought, and sophistication of online communication strategies, virtually all campaigns reviewed included content in their patient testimonials and narratives reinforcing common themes inherent to the struggle of terminally ill patients seeking expanded access. Although a full qualitative content analysis of online campaign data is beyond the scope of this article, the content generally grouped into themes of: a) rapid deterioration of patient’s health indicating an immediate and urgent need for experimental treatment; b) lack of any alternative treatment options and/or exhausting all available treatment options; c) description of the devastating impact of the condition on the patient’s family in order to encourage public support; d) identifying a drug manufacturer (and in some cases the FDA) as the primary barrier for lack of access; and e) overwhelmingly “positive” characterization of experimental drugs petitioned (including characterizing an experimental drug as a “wonderdrug”). Collectively, online campaigns appeared to reinforce and propagate a common and shared narrative among terminally ill patients seeking expanded access: that no other treatment options are available; that patients are being denied access to treatment; and experimental drug access represents their last and best hope for a life-saving intervention.

### Limitations

This study has certain limitations that may impact the validity and generalizability of results in relation to expanded access online patient behavior. Specifically, the results are limited by the sampling and search methodology used in the study, which relied on a combination of keyword queries on a popular search engine and using in-site searches directly on online petition and social media platforms. These queries return non-random search results prioritizing content based on the search engine or website platform’s own propriety algorithm, which determines the relevance to the topic and may not be comprehensive. Website sampling was also limited to a specific point in time and five related search terms. Additionally, we did not pursue a formal in-depth coding or qualitative analysis of content contained on online petitions and social media sites to more appropriately identify themes associated with the topic. Hence, these factors may affect the generalizability of results and limits some of the assumptions made by authors.

## Discussion

This review of the expanded access digital environment indicates that although there is a mix of drug candidates, firms petitioned, social media strategies, and treatment access outcomes, one particular trend stands out: the majority of expanded access online petitions and social media campaigns involve cancer patients and their families. This may not be particularly surprising given that an estimated 589,000 Americans are expected to die from cancer in 2015, making it the second most common cause of death in the country [69]. Additionally, the narratives included in the vast majority of case studies examined indicate that petitions and social media are used out of desperation, as patients have exhausted conventional treatment options, including enrolling in manufacturer clinical trials. Additionally, the regulatory and policy environment for expanded access leaves much to be desired, as manufacturers continue to lack needed incentives to more uniformly develop EAPs, patients are often unaware of expanded access pathways or how to navigate them, and “right-to-try” laws have their own inherent legal and practical challenges.

From the patient advocacy perspective, a review of online campaigns indicates a wide variation in levels of sophistication and engagement with equally inconsistent outcomes for gaining access to treatment. Some campaigns appear to be professionally prepared marketing presentations with attractive visuals and articulately worded summaries outlining the arduous journeys of terminally-ill patients and families. Others are less polished, containing only basic information describing the patient’s condition and lacking sufficient detail as to what action the public can take to help them access experimental drugs. What makes a “successful” expanded access campaign ultimately remains uncertain despite our review, given the small sample size of cases reviewed, unique circumstances of each patient, and the uncertain outcome based on whether access was actually achieved. However, campaigns observed with the most Change.org signatures and/or social media interaction/presence also appear to have garnered substantial media coverage from popular news outlets that have helped further their cause (e.g. CNN, The Today Show).

Given the high level of variability and uncertain benefit of patient use of online petitions and social media, combined with the challenges faced by the FDA, manufacturers, and state governments in improving expanded access through regulation and public policy, stakeholders should begin the process of exploring policy changes at the federal level that can improve patient education and facilitate more efficient exploration of available options for pursuing expanded access. Policy changes should focus on developing transparent, equitable, and standardized processes, which balance the competing needs of terminally ill patients with the reality of commercial pressures, possibility of poor drug-to-candidate fit, and scarce availability of experimental drugs. Currently, terminally ill patients are left to navigate a confusing patchwork of manufacturer EAPs, recently enacted state “right-to-try” laws, FDA’s revised expanded access pathway, and various information sources online. This confusion regarding access options to experimental drugs largely emanates from the fact that manufacturers are not transparent about their expanded access policies and their specific parameters for approving requests.

In response, the proposed 2015 Andrea Sloan Compassionate Use Reform and Enhancement (CURE) Act (H.R.909, 114th Congress), named after an ovarian cancer patient who died in 2014, would have amended the Federal Food, Drug, and Cosmetic Act to require companies to publicly disclose corporate expanded access policies for certain investigational drugs. The majority of the provisions of the CURE Act were then incorporated into legislation approved by the U.S. House of Representatives in July 2015 known as the 21st Century Cures Act (H.R.6, Title II, Subtitle E), a highly touted piece of bipartisan legislation aimed at modernizing and accelerating the discovery, development, and delivery of life-saving drugs and therapy. The incorporated legislation (collectively referred to as “CURE Act Provisions”) would require companies to: 1) establish a procedure and single point-of-contact for expanded access requests; 2) explain the general criteria for sponsor’s approval of such requests; and 3) disclose the amount of time anticipated to respond to requests [70]. Conspicuously absent from the CURE Act Provisions was a requirement in the original CURE Act requiring companies to provide patients with a written notice explaining a denial of a request [71].

Policy reforms such as the CURE Act Provisions could provide patients with critical information to better assess the utility of social media engagement based upon the presence/absence and specific terms of a sponsor’s compassionate use/expanded access policies. The availability of such information might preclude the need for individuals to pursue direct advocacy that is unstructured, time-intensive, and of questionable impact. Specifically, the current lack of commitment by manufacturers to proactively create expanded access policies or EAPs is a significant barrier to patient education and treatment access. Further, although a search for the term “expanded access” on the National Institutes of Health (NIH) website ClinicalTrials.gov yielded more than 300 results (including active, closed, and finished studies) of expanded access programs and protocols, navigating these results can be unwieldy for patients and clinicians, and many pharmaceutical firms do not actively publicize their EAPs. The CURE Act Provisions were designed to specifically address this limitation by making disclosure of expanded access policies a condition of FDA investigational drug approval. By requiring disclosure as a condition for an IND, the provisions could act as a catalyst for robust expanded access policy creation, disclosure, and could also lead to some needed industry standardization in response to the growing patchwork of state “right-to-try” laws.

The original CURE Act also required the Secretary of Health and Human Services to establish an Expanded Access Task Force to “explore mechanisms for improving the access of individual patients have to investigational drugs …” [71]. Specifically, the duties of this task force would include exploring possible incentives for firms to approve expanded access requests and evaluating ways to streamline and standardize processes of submitting requests by patients/clinicians. Revisiting the creation of such a task force is worth merit as lack of financial incentives appears to be a significant barrier to expanded access approval by sponsors [18, 35, 47]. Designing financial incentives could be modeled based upon existing incentives available under the Orphan Drug Act of 1983 (ODA), if not already available for cancers related to rare or orphan diseases [51]. This could include providing tax credits for clinical trial costs associated with only intermediate-sized expanded access INDs or protocols, to encourage consolidation of expanded access requests, collection of potential data on adverse events, and streamline approval processes for manufacturers. In all cases, any form of financial incentives associated with expanded access programs should be appropriately structured to: 1) target smaller/mid-sized firms that lack resources to develop EAPs; 2) prioritize expanded access drug candidates with high potential to meet the needs of an underserved population without any viable treatment options; 3) balance financial incentives with the currently poorly regulated price of access to experimental treatment for patients; and 4) prioritize expanded access when responding to serious public health emergencies (e.g. the 2013 MenB outbreak, 2014 Ebola outbreak, combating tuberculosis and anti-microbial resistance, and use for opioid overdose intervention) [18, 20, 46, 72–74].

Finally, efforts should be made to pragmatically simplify the process for patients to search for and submit expanded access requests to EAPs and the FDA. This could include the development of a centralized database that houses all expanded access policies and links directly to pharmaceutical manufacturer/sponsor EAPs, as mandated to be disclosed under the CURE Act Provisions. The database could also incorporate data already available from ClinicalTrials.gov linking to existing expanded access programs and protocols, which patients could then directly access if qualified. Other patient advocacy stakeholders (such as the American Cancer Society, which operates a free and confidential Clinical Trials Matching Service) could then use these resources to provide services to help patients and their clinicians match EAPs and expanded access programs/protocols that are appropriate for each individual’s unique medical and personal circumstances. Manufacturers and industry trade groups, such as the Biotechnology Industry Organization and the Pharmaceutical Research and Manufacturers of America that have expressed commitment to improving expanded access pathways, should also aid in the creation of this information source in partnership with patient advocacy groups and the FDA [56].

## Conclusions

Regardless of the medium, use of online petitions and social media platforms by terminally ill patients in the hope of gaining access to experimental treatment is sure to continue in a growing “digital” and “social” health landscape. Importantly, this form of digital patient advocacy appears to be a symptom of current policy fragmentation between the FDA, individual states, industry, and patient advocacy groups, as well as the absence of reliable information sources needed for patients when assessing whether expanded access pathways are viable options in the face of often serious and terminal diseases. Hence, lessons learned from the case studies reviewed and the policy environment examined provides a compelling reason to explore proactive reform of current expanded access policies. This should include taking advantage of heightened attention to the issue headlined by the CURE Act Provisions contained in the 21st Century Cures Act, though the legislation faces its own uncertain future as the U.S. Senate takes it up in 2016. While expanded access may not be the best option for the vast majority of individuals, comprehensive, accurate, and easily accessible information detailing expanded access options will go a long way to help terminally ill patients and their families make informed choices on the utility of pursuing an online petition or social media campaign in the hope of gaining access to experimental drugs.

## Additional file

Additional file 1: Table S1. Compassionate use online petition and social media campaigns. (DOCX 33 kb)
