# Supplementary material for: Going “social” to access experimental and potentially life-saving treatment: an assessment of the policy and online patient advocacy environment for expanded access
Source: BMC Med. 2016 Feb 2;14:17. doi: 10.1186/s12916-016-0568-8 (PMC4739083; doi:10.1186/s12916-016-0568-8)
Supplement: Additional file 1: Table S1. — Compassionate use online petition and social media campaigns. (DOCX 33 kb) [file 12916_2016_568_MOESM1_ESM.docx]

**Additional file 1: Table S1** Compassionate use online petition and social media use campaigns

| **Name** | **Date petition created** | **Disease** | **Self-reported outcome** | **Campaign type** | | **Drug characteristics** | | |
| --- | --- | --- | --- | --- | --- | --- | --- | --- |
|  |  |  |  | **Online petition website  (number of signatures)** | **Social media platforms** | **Drug candidate name** | **Companies petitioned** | **FDA market authorization; drug name; approved indications** |
| Patient A | 2013 | Stage IV melanoma | Entered into trial through normal channels, but soon disqualified due to complications. Did not receive expanded access | Change.org (525,738 signatures^a^) | Facebook (cause page), Twitter account, and personal website | Anti-PD-1 | Bristol-Myers Squibb and Merck | Approved (Merck, Sept 2014); pembrolizumab (trade name Keytruda); metastatic non-small cell lung cancer |
| Patient B | 2013 | Lung cancer (Pancoast tumor) | Unable to verify | Change.org (448 signatures^a^) | N/A | Anti-PD-1 | Bristol-Myers Squibb and Merck | Approved (Merck, Sept 2014); pembrolizumab (trade name Keytruda); metastatic non-small cell lung cancer |
| Patient C | 2013 | Stage IV melanoma | Did not receive access | Change.org (30,311 signatures^a^) | N/A | Anti-PD-1 | Merck | Approved (Merck, Sept 2014); pembrolizumab (trade name Keytruda); metastatic non-small cell lung cancer |
| Patient D | Dec 2013 | Anaplastic  medulloblastoma | Did not receive access | The U.S. White House online petition (102,215 signatures) | Facebook (community page), Twitter account, and YouTube video | ANP | Request directly to FDA | Not approved |
| Patient E | 2013 | Diffuse intrinsic pontine glioma (DIPG) brainstem tumor | Received access | Change.org (19,992 signatures^a^) | N/A | ANP | FDA approved under single patient protocol | Not approved |
| Patient F | 2013 | Gastric cancer | Unknown | Change.org (103,179 signatures^a^) | N/A | Ramucirumab | Eli Lilly | Approved (Eli Lilly, Apr 2014); ramucirumab (trade name Cyramza); advanced gastric or gastro-esophageal junction adenocarcinoma |
| Patient G | 2011 | Stage IV melanoma | Did not receive access | Change.org (200,701 signatures^a^) | Facebook (community page), Twitter account, YouTube video, and blog (WordPress) | Anti-PD-1 (BMS-936558) | Bristol-Myers Squibb | Not approved^b^ |
| Patient H | Care2.com created Oct 2014 | MPS II Hunter syndrome | Did not receive access because of exclusion criteria | Care2.com (62,041 signatures) | Facebook (community page), YouTube video, and blog (WordPress, CaringBridge) | Enzyme replacement | Shire | Not approved |
| Patient I | Diagnosed Oct 2005 | Breast cancer | Received treatment of experimental drug, but switched to other treatment | N/A | Facebook (public figure page), YouTube video, personal website, and affiliated non-profit website | Pertuzumab | Genentech | Approved (Genentech [acquired by Roche], Jun 2012); Pertuzumab (trade name Perjeta); HER2-positive metastatic breast cancer |
| Patient J | 2013 | Coxsackievirus | Unknown | Change.org (1,465 signatures^a^) | N/A | Pleconaril | Merck | Not approved |
| Patient K | 2013 | Treatment of malignant rhabdoid tumor of the kidneys that resulted in adenovirus | Received treatment | Change.org (19,786 signatures^a^) | Facebook (community page), Twitter hashtag, Tumblr, and CaringBridge | Brincidofovir (CMX001) | Chimerix | Not approved |
| Patient L | 2014 | Kidney cancer | Did not receive access | Change.org (481,795 signatures) | Facebook (removed), Twitter account, and blog (WordPress) | Anti-PD-1 (MK-3475) | Merck | Approved (Merck, Sept 2014); pembrolizumab (trade name Keytruda); metastatic non-small cell lung cancer |
| Patient M | 2013 | Diffuse intrinsic pontine glioma (DIPG) | Received treatment | Change.org (68,714 signatures^a^) | Facebook (community page) and YouTube video | ANP | FDA compassionate use petition | Not approved |
| Patient N | 2013 | Mantle cell lymphoma | Unknown | Change.org (1,303 signatures^a^) | N/A | Ibrutinib (PCI-32765) | Pharmacyclics | Approved (Pharmacyclics, Nov 2013); ibrutinib (trade name Imbruvica); mantle cell lymphoma (2013), chronic lymphocytic leukemia (2014), and Waldenstrom macroglobulinemia (2015) |
| Patient O | 2013 | Stage IV melanoma | Unknown | Change.org (1,171 signatures^a^) | N/A | Anti-PD-1  (widen MK3475 expanded access) | Merck | Approved (Merck, Sept 2014); pembrolizumab (trade name Keytruda); metastatic non-small cell lung cancer |
| Patient P | Blog created 2010 | Brain tumor (suspected, but not proven to be medulloblastoma) | Received treatment | N/A | YouTube video and blog (WordPress) | GDC-0449 | Genentech | Approved (Genentech [acquired by Roche], Jan 2012); vismodegib (trade name Erivedge); metastatic or recurrent locally advanced basal cell carcinoma |
| Patient Q | 2012 | Teratoma (brain tumor) | Received treatment | Change.org (3,765 signatures^a^) | Facebook (community page) and CaringBridge | CDK/patient’s family self-reported the drug as palbociclib | Pfizer | Approved (Pfizer, Feb 2015); palbociclib (trade name Ibrance); ER-positive and HER2-negative metastatic breast cancer |
| Patient R | 2012 | Spinal muscular atrophy | Began ISIS-SMNRx randomized trial 9 Nov 2015 | Change.org (13,421signatures^a^) | Facebook (community page) | ISIS-SMNRx | ISIS Pharmaceuticals Inc. | Not approved |
| Patient S | 2013 | Stage IIIC ovarian cancer | Received treatment from competitor company | Change.org (234,837 signatures^a^) | Facebook (community page) and Twitter account | PARP inhibitor BMN 673 | BioMarin Pharmaceutical Inc. | Not approved |
| Patient T | 2015 | Amyotrophic lateral sclerosis (ALS) | Did not receive access | Change.org (4,519 signatures) | N/A | **NurOwn**™ **(autologous, adult stem cell therapy)** | **BrainStorm Cell Therapeutics** | Not approved |
| Patient U | Diagnosed Apr 2012 | Stage IV alveolar soft part sarcoma (ASPS) | Did not receive access | N/A | Facebook (community page), Twitter account, YouTube video, blog (WordPress, CaringBridge), and website | Anti-PD-1 | Genentech, Bristol-Myers Squibb, and Merck | Approved (Merck, Sept 2014); pembrolizumab (trade name Keytruda); metastatic non-small cell lung cancer |
| Patient V | 2013 | Kidney cancer | Received treatment, but discontinued due to side effects | Change.org (17,125 signatures^a^) | Blog (blogspot) | Anti-PD-1/L1 | Bristol-Myers Squibb, Merck, MedImmune, CureTech, and Genentech | Approved (Merck, Sept 2014); pembrolizumab (trade name Keytruda); metastatic non-small cell lung cancer |
| Patient W | 2013 | Metastatic breast cancer | Did not receive access (requested for off-label use) | Change.org (1,275 signatures^a^); MoveOn.org (81 signatures) | N/A | Xofigo (radium-223) | Bayer HealthCare Pharmaceuticals | Approved (Bayer, May 2013); **radium**-223 dichloride (trade name Xofigo); patients with castration-resistant prostate cancer, symptomatic bone metastases, and no known visceral metastatic disease |

Data updated as of 30 November 2015. Online petitions reviewed: avaaz.org; care2.com; causes.com; change.org; ipetitions.com; front.moveon.org; thepetitionsite.com; and the U.S. White House’s online petition site. Social media platforms reviewed: Facebook; Twitter; YouTube; Tumblr; CaringBridge; blog sites (e.g. WordPress); and other linked websites. ^a^Closed/completed petition; ^b^the Bristol-Myers Squibb candidate did not receive approval, but a similar anti-PD-1 drug pembrolizumab (trade name Keytruda), developed by Merck, was approved by the FDA in September 2014. ANP, antineoplaston; N/A, not available
